# The impact of physical activity on social anxiety among college students: an analysis of the chain mediation effect of family support and self-efficacy

**DOI:** 10.7717/peerj.20511

**Published:** 2026-01-05

**Authors:** Baiyi Yang, Xiaodi Yang, Zhengyang Fan, Chang Liu, Zhanfei Zheng

**Affiliations:** 1 College of Sports Science, Southwest Jiaotong University, Chengdu, Sichuan, China; 2 College of Sports Science, Cangzhou Normal University, Cangzhou, Hebei, China; 3 Department of Sports Science, Wenzhou Medical University, Wenzhou, Zhejiang, China; 4 Sports Coaching College, Beijing Sport University, Beijing, China

**Keywords:** Physical activity, Social anxiety, Family support, Self-efficacy, Mediation effect

## Abstract

**Background:**

This study aimed to explore the mechanisms through which physical activity affects social anxiety in college students, with a specific focus on the sequential mediating roles of family support and self-efficacy.

**Methods:**

A total of 391 valid responses were analyzed using the Physical Activity Rating Scale (PARS-3), the Interaction Anxiousness Scale (IAS). Family Support Scale (PSS-Fa), and the General Self-Efficacy Scale (GSES). Mediation analyses were conducted with structural equation modeling (SEM) and bootstrapping procedures (5,000 resamples).

**Results:**

Physical activity significantly predicted lower social anxiety (β =  − 0.187, *p* < .001). Both family support (β =  − 0.309, *p* < .001) and self-efficacy (β =  − 0.390, *p* < .001) mediated this association, with the sequential pathway (β =  − 0.073, *p* < .001) accounting for 13.35% of the total effect.

**Conclusion:**

Physical activity was associated with lower social anxiety indirectly *via* higher family support and self-efficacy, while the direct effect was not statistically significant in the full mediation model. These findings provide empirical support for incorporating physical activity into university-based mental health strategies aimed at alleviating social anxiety.

## Introduction

Social anxiety refers to a persistent fear or intense apprehension about being negatively evaluated during social interactions, and it is particularly prevalent among college students who are navigating significant social and academic transitions (Association, 2013; Schlenker & Leary, 1982). Students experiencing social anxiety often face challenges in maintaining healthy interpersonal relationships, achieving academic success, and preserving psychological well-being (Stein & Stein, 2008). Given the heightened social expectations and rigorous academic demands of university life, college students represent a population especially susceptible to developing symptoms of social anxiety (Eisenberg et al., 2007).

Numerous studies have demonstrated that physical activity provides substantial mental- health benefits, including reductions in anxiety and depression (Mammen & Faulkner, 2013; Rebar et al., 2015). In exercise psychology, partivipantion in physical activity engages cognitive, emotional, and behavioral behavioral processes that extend benefits beyond physical fitness (Biddle & Mutrie, 2008; Mandolesi et al., 2018). Among college students, regular participation is associated with better social connectedness, improved emotion regulation, and more effective coping with stress (Craft, 2005; Ekkekakis, 2013). Recent syntheses sharpen both the magnitude and pathways of the physical activity–anxiety link in student and adult populations. An umbrella review of 97 reviews concluded that physical activity confers clinically meaningful reductions in anxiety and psychological distress across diverse adult groups (Singh et al., 2023). In university samples specifically, a recent meta-analysis reported that exercise interventions reduce anxiety among college students (Chen et al., 2025). Mechanistically, a 2024 systematic review of mediators/moderators highlights self-efficacy as a plausible pathway through which activity benefits mental health (White et al., 2024). Complementing intervention evidence, longitudinal and dose–response analyses indicate that higher habitual activity is associated with lower incident anxiety (Li et al., 2025). At the same time, cohort findings are not uniformly positive (*e.g.*, null associations in some developmental windows), underscoring heterogeneity and the value of theory-driven mediator tests (Cordoba-Grueso, Galaviz & Parker, 2024). These updates collectively motivate our focus on family support and self-efficacy as candidate mechanisms in college students.

Two critical psychosocial factors, family support and self-efficacy, have recently gained attention as potential mediators in the relationship between physical activity and social anxiety. Within university settings, recent reviews reaffirm that stronger social support is associated with lower anxiety and better psychological adjustment, reinforcing its relevance as a protective factor in student mental health (Vicary et al., 2025). Parallel student-focused work indicates that physical activity is positively related to general self-efficacy and may reduce anxiety through improved efficacy beliefs and related resources. Family support encompasses emotional encouragement, tangible assistance, and a sense of belonging, all of which serve as protective resources for mental health (Milošević & Tubić, 2018; Rueger, Malecki & Demaray, 2010). Stronger family support has been consistently linked to higher engagement in physical activity and better psychological outcomes (Anderson-Butcher et al., 2018). Self-efficacy, defined as an individual’s belief in the capability to manage tasks and challenges, bolsters resilience and reduces vulnerability to anxiety (Bandura, 1997). This pattern is consistent with evidence that self-efficacy is closely tied to adaptive adjustment in health and exercise contexts (Luszczynska, Scholz & Schwarzer, 2005; McAuley et al., 2011). Guided by Social Cognitive Theory, supportive family contexts cultivate self-efficacy through mastery experiences, vicarious learning, verbal persuasion, and the regulation of affective states, while simultaneously strengthening emotional security and belonging (Bandura, 1997). These experiences are internalized as stronger self-efficacy, which more proximally shapes anxiety-related outcomes. In university settings, participation in physical activity can catalyze supportive family interactions, for example by sharing progress and routines, thereby elevating perceived family support and subsequently promoting self-efficacy. Together, these mechanisms predict lower social anxiety. Accordingly, we pre-specify a sequential pathway, physical activity → family support → self-efficacy → social anxiety, and test this chain mediation in the present study (McAuley et al., 2011; Rueger, Malecki & Demaray, 2010).

This study advances prior work by testing a sequential mediation pathway (family support → self-efficacy) linking physical activity to social anxiety. The specification reflects theory that family support provides resources that foster self-efficacy, which supports adaptive emotion regulation and lowers anxiety. We therefore model a directional path from family support to self-efficacy and allow the mediators to correlate. We pre-specify:

H1. Physical activity is negatively associated with social anxiety.

H2. Family support mediates the association between physical activity and social anxiety.

H3. Self-efficacy mediates the association between physical activity and social anxiety.

H4. Chain mediation: physical activity → family support → self-efficacy → lower social anxiety.

## Materials & Methods

### Participants

This research examined the relationship between physical activity and social anxiety among college students, specifically testing the mediating roles of family support and self-efficacy. Using stratified random sampling across undergraduate and graduate cohorts at multiple universities in Sichuan Province, China, we administered an anonymous online questionnaire that was proportionally distributed across demographic strata. Of the 399 questionnaires issued, 391 valid responses remained after excluding patterned, incomplete, or implausibly rapid submissions, yielding an effective response rate of 97.99% (Table 1). The achieved sample (*N* = 391) is supported by structural equation modeling (SEM) and bootstrapped mediation recommendations; post-hoc sensitivity (α = .05, 1–β = .80) indicates adequate power to detect small effects (min —r—≈0.14). All study instruments were self-administered *via* an anonymous online survey that participants completed on their own devices and at a time and location of their choosing, without in-person proctoring or classroom scheduling.

The study protocol was approved by the Ethics Committee of Southwest Jiaotong University (Approval No: SWJTU-2406-QT (176)) and adhered to the Declaration of Helsinki. Before any survey items appeared, participants viewed an ethics-committee–approved information sheet and provided electronic informed consent by checking an explicit agreement box; no physical signatures were obtained. No direct identifiers (*e.g.*, name, email, phone, student ID) were collected, and IP addresses or device identifiers were not stored. The dataset exported from the platform contained no identifiers; each record was assigned a random numeric code and stored on a password-protected, institution-managed server with access restricted to the research team. All analyses were performed on the de-identified dataset, and the de-identified raw measurements are available in the Supplementary File.

### Research instruments

#### Physical Activity Rating Scale (PARS-3)

Physical activity over the past month was measured with the Physical Activity Rating Scale (PARS-3) (Liang, 1994), which captures intensity, duration, and frequency on a 5-point response format. The total score is computed as intensity × (duration−1) × frequency, with established cut-points for low (≤19), moderate (20–42), and high (≥43) activity. The PARS-3 has demonstrated content and construct validity in Chinese student populations and has been widely used in epidemiological and educational settings in China. In the present study, internal consistency was acceptable (Cronbach’s α = 0.812). In addition to reliability, we considered validity in two ways: (1) theoretical/construct validity based on the established three-component structure and conventional scoring rule, and (2) convergent/criterion considerations insofar as higher PARS-3 scores showed the expected direction of associations with psychosocial outcomes (lower social anxiety; higher family support and self-efficacy) in bivariate analyses (see ‘Results’). Together, these points align with prior reports of PARS-3 validity and support its use in this population.

**Table 1:** Distribution of demographic variables among survey respondents.

Variable	Category	Number of people	Percentage %
Gender	Male	184	47.06
	Female	207	52.94
Education background	Regular college course	223	57.03
	Master and PhD	168	42.97
Age	20 and below	139	35.55
	21–23	143	36.57
	24 and above	109	27.88
Total		391	100

#### Social anxiety scale

Social anxiety was assessed using the Interaction Anxiousness Scale (IAS), originally developed by Leary and adapted to Chinese contexts by Peng, Gong & Zhu (2004). The IAS contains 15 items measuring two dimensions: subjective anxiety without explicit external behaviors, and anxiety in unexpected social contexts. Responses were scored on a 5-point Likert scale (1 = strongly disagree, 5 = strongly agree), with reverse scoring applied to items 3, 6, 10, and 15. Total scores ranged from 15 to 75, with higher scores indicating greater social anxiety. The IAS showed high internal consistency (Cronbach’s α = 0.917) and satisfactory confirmatory factor analysis indices (*χ*2/*df* = 3.207, RMSEA = 0.075, GFI = 0.904, CFI = 0.935, TLI = 0.923, SRMR = 0.085).

#### Family support scale

The PSS-Fa, originally developed by Procidano & Heller (1983) and adapted for Chinese samples, comprises 15 dichotomous items (Yes = 1, No = 0), with four reverse-scored items (3, 13, 14, 15). We computed (a) a composite sum score (range 0–15; higher values indicate greater perceived family support) and (b) a normalized proportion (sum/15; range 0–1) for ease of interpretation in descriptive analyses. For clarity, the reference value 0.5 on the proportion scale equals 7.5/15 on the sum score; we indicate which metric (raw *vs.* scaled) is used in each table and analysis. Internal consistency in the present sample was acceptable (Cronbach’s α = 0.802).

#### Self-efficacy scale

The Chinese version of the 10-item General Self-Efficacy Scale (GSES) (Caikang, 2001) was administered. Consistent with validation studies of the Chinese adaptation and prevailing practice, the scale was treated as unidimensional, and the total score was used in all analyses. Although alternative factor solutions have been reported in some localized validations, extensive psychometric work supports a single general self-efficacy factor with stable properties across cultures (Luszczynska, Scholz & Schwarzer, 2005). Internal consistency in the present sample was excellent (Cronbach’s α = 0.94).

### Statistical methods

Data were managed and initially screened in Excel, and analyses were conducted in SPSS 26.0 and IBM AMOS 26.0. Potential common-method variance was evaluated using Harman’s single-factor test: all observed items from the PARS-3, IAS, PSS-Fa, and GSES were entered into an unrotated principal-components analysis; we report the Kaiser–Meyer–Olkin (KMO) statistic and Bartlett’s test, the number of eigenvalues >1, the variance explained by the first factor, and the cumulative variance. A first-factor share <40% was interpreted as indicating no serious common-method bias. Descriptive statistics and Pearson correlations were computed. The a priori serial mediation (physical activity → family support → self-efficacy → social anxiety) was tested using PROCESS v3.4 and SEM in AMOS; alternative parallel specifications were acknowledged but not estimated. Both PROCESS and SEM models controlled for gender, age, and education. In the SEM, these covariates were specified as observed exogenous variables with direct paths to family support, self-efficacy, and social anxiety; global fit indices and the direction/significance pattern of the focal paths were comparable to the model without covariates. Distributional assumptions (univariate skewness and kurtosis), linearity, and homoscedasticity of residuals were examined. For all direct and indirect effects, bias-corrected bootstrapped 95% confidence intervals (5,000 resamples) were used, which are robust to non-normality and heteroscedasticity; standardized coefficients are reported. As a sensitivity analysis, Spearman correlations yielded inferences consistent with the Pearson estimates.

## Results

### Common method bias test

Harman single-factor test. The data were suitable for factor analysis (KMO = 0.906; Bartlett’s *χ*^2^(903) = 11,399.17, *p* < .001). Nine factors with eigenvalues >1 were extracted. The first factor explained 33.89% of the total variance (cumulative variance across the first five factors = 56.90%), which is below the 40% threshold, suggesting that common-method bias is unlikely to threaten the findings (see Table 2 for the full summary).

**Table 2:** Harman single-factor test summary.

**Metric**	**Value**
Sample size (*N*)	391
Item set	PARS-3, IAS, PSS-Fa, GSES
KMO	0.906
Bartlett’s test of sphericity	*χ*^2^(903) = 11,399.17, *p* < .001
Extraction (unrotated)	Principal components
Number of factors with eigenvalues >1	9
% variance explained by 1st factor	33.89%
Cumulative % variance (first 5 factors)	33.89%, 42.30%, 48.95%, 53.38%, 56.90%

**Notes.**

1 Harman’s single-factor procedure was applied to the full set of observed items across all study instruments to screen for common-method variance.

2 A first-factor variance proportion <40% is commonly taken to indicate an absence of serious common-method bias in self-report surveys.

3 Values correspond to the dataset analyzed in the manuscript; all mediation inferences were updated to avoid implying a significant direct effect when mediators are included.

Given the potential influence of factors such as the measurement environment, questionnaire instructions, and survey context, common method bias may exist in data collected through self-report questionnaires (Podsakoff et al., 2003). (1) Descriptive statistics: A one-sample *t*-test revealed that the mean scores for physical activity (*M* = 3.30, SD = 0.97), social anxiety (*M* = 3.21, SD = 0.92), and self-efficacy (*M* = 3.48, SD = 0.98) were all significantly higher than their respective median reference values (*p* < 0.01), indicating generally high levels of physical activity and self-efficacy, as well as a moderate level of social anxiety among participants. For the PSS-Fa, we report the scaled proportion score (raw total ÷ 15) unless otherwise noted: *M* = 0.60, SD = 0.25, indicating moderate-to-high perceived family support among participants. For completeness, the corresponding raw total is *M* = 9.0, SD = 3.8 (0–15). All subsequent mediation models used standardized variables and are invariant to this reporting choice. (2) Correlation analysis: Pearson correlation analysis showed that physical activity was significantly negatively correlated with social anxiety (*r* = −0.547, *p* < 0.01). In addition, physical activity was positively correlated with both family support (*r* = 0.532, *p* < 0.01) and self-efficacy (*r* = 0.520, *p* < 0.01), while family support and self-efficacy were also positively correlated (*r* = 0.530, *p* < 0.01). (3) Mediation analysis: Structural equation modeling (SEM) confirmed the mediating effects of family support and self-efficacy on the relationship between physical activity and social anxiety. The total indirect effect was −0.366 (95% CI [−0.441, −0.296]), accounting for 66.86% of the total effect. Specifically, the following mediation pathways were identified: Path 1: Physical activity → Family support → Social anxiety (β = −0.165, 30.16% of the total effect); Path 2: Physical activity → Self-efficacy → Social anxiety (β = −0.128, 23.4% of the total effect); Path 3 (Chain Mediation): Physical activity → Family support → Self-efficacy → Social anxiety (β = −0.073, 13.35% of the total effect).

**Table 3:** Single-sample *T*-test for physical activity, social anxiety and self-efficacy.

Variable	M	SD	T
Physical activity	3.30009	0.970668	6.113^**^
Social anxiety	3.21455	0.918887	4.617^**^
Self-efficacy	3.47826	0.980248	9.648^**^

**Notes.**

* *p* < 0.05.

** *p* < 0.01.

**Table 4:** Single-sample *T*-test for family support.

Variable	M	SD	T
Family support	0.59847	0.251192	7.751^**^

**Notes.**

* *p* < 0.05.

** *p* < 0.01.

### Statistics of college students’ current physical activity, social anxiety, family support and self-efficacy

Of the 399 questionnaires distributed, 391 valid responses were included in the analysis. For the scales of physical activity, social anxiety, and self-efficacy, one-sample t-tests were conducted using a test value of 3, which represented the midpoint of the respective scales (see Table 3). The results indicated that college students’ scores on physical activity, social anxiety, and self-efficacy were all significantly higher than the scale midpoint, suggesting generally favorable development in these domains among the participants. For the family support scale, which was scored dichotomously (0 = no support, 1 = support), a one-sample *t*-test was performed using a test value of 0.5 (the scale mean) as the reference (see Table 4). Results showed that family support scores were significantly higher than 0.5, indicating a high perceived level of family support among the surveyed college students.

### Correlation analysis of each variable

Pearson correlation analysis was conducted to examine the relationships among physical activity, social anxiety, family support, and self-efficacy in college students. Physical activity, family support, and self-efficacy were each significantly negatively correlated with social anxiety, and significant positive intercorrelations were observed among physical activity, family support, and self-efficacy (Table 5).

Specifically, as shown in Table 5, physical activity was negatively associated with social anxiety (*r* = −0.547, *p* < 0.01). Physical activity was positively associated with family support (*r* = 0.532, *p* < 0.01) and with self-efficacy (*r* = 0.520, *p* < 0.01), and family support was positively associated with self-efficacy (*r* = 0.530, *p* < 0.01). These associations are consistent with the hypothesized mediation pathways while remaining agnostic about causal direction given the cross-sectional design. The significant positive correlation between family support and self-efficacy (*r* = 0.530, *p* < 0.01) suggests that family support plays an important role in enhancing individuals’ self-confidence and sense of competence, consistent with previous research. Further analysis confirmed that physical activity directly reduced social anxiety among college students (β = −0.187, *p* < 0.001), and also exerted indirect effects through family support (β = −0.309, *p* < 0.001) and self-efficacy (β = −0.390, *p* < 0.001). The chain mediation effect accounted for 13.35% of the total effect, highlighting the synergistic contributions of familial environment and psychological resilience. Although the conclusions are limited by the cross-sectional design and regional sample, these findings provide preliminary theoretical support for campus-based mental health interventions. Future randomized controlled trials are warranted to further validate the efficacy of physical activity programs in alleviating social anxiety.

### Mediating effect test

Taking physical activity as the independent variable, social anxiety as the dependent variable, and family support and self-efficacy as mediators, a chain mediation analysis was conducted using SPSS 26.0 and the PROCESS 3.4 plug-in, while controlling for demographic variables such as gender, educational background, and age. The specific steps and results are presented in Table 6. In the first step, social anxiety was entered as the dependent variable, with gender, educational background, and age as covariates to control their potential influences on college students’ social anxiety. In the second step, physical activity was added to the regression model to evaluate its total effect on social anxiety after controlling for covariates. Results showed that physical activity significantly and negatively predicted social anxiety (β = −0.552, *p* < 0.001), supporting hypothesis H1. In the third step, family support and self-efficacy were sequentially introduced into the regression model to test their mediating roles between physical activity and social anxiety, and to assess the presence of a chain mediating effect. The results indicated that physical activity significantly and negatively predicted social anxiety (β = −0.187, *p* < 0.001). Both family support (β = −0.309, *p* < 0.001) and self-efficacy (β = −0.390, *p* < 0.001) negatively predicted social anxiety. Additionally, physical activity significantly and positively predicted family support (β = 0.531, *p* < 0.001) and self-efficacy (β = 0.327, *p* < 0.001). Furthermore, family support significantly and positively predicted self-efficacy (β = 0.355, *p* < 0.001). The regression coefficients clearly demonstrated that family support and self-efficacy exerted a significant chain mediation effect in the relationship between physical activity and social anxiety, thus supporting hypotheses H2 and H3. The mediating effect model was further validated using AMOS 26.0, showing good model fit indices: *χ*^2^/*df* = 3.346, CFI = 0.978, TLI = 0.954, RMSEA = 0.044, SRMR = 0.039. When the SEM was re-estimated controlling for gender, age, and education, global fit and the direction/significance of the hypothesized paths remained essentially unchanged. Given the a priori, theory-driven direction from family support to self-efficacy, the serial specification was retained; parallel variants were acknowledged but not estimated.

**Table 5:** Correlation analysis of physical activity, family support, self-efficacy and social anxiety.

Variable	1	2	3	4
Physical activity	1			
Family support	0.532^**^	1		
Self-efficacy	0.520^**^	0.530^**^	1	
Social anxiety	−0.547^**^	−0.611^**^	−0.646^**^	1

**Notes.**

* *p* < 0.05.

** *p* < 0.01; same below.

The number in the first row represents the variable ordinal number.

1 Physical Activity.

2 Family Support.

3 Self-Efficacy.

4 Social Anxiety.

**Table 6:** Mediating effect regression model of family support and self-efficacy.

Variable	Social anxiety		Family support		Self-efficacy		Social anxiety	
	β	t	β	t	β	t	β	t
Gender	0.027	0.530	0.011	0.223	0.029	0.576	0.053	1.537
Age	0.048	0.835	−0.059	−1.029	−0.158	−2.783	−0.045	−1.146
Education background	0.020	0.341	0.062	1.072	−0.019	−0.343	0.041	1.047
Physical activity	−0.552	−12.964	0.531	12.284	0.327	6.889	−0.187	−4.314
Family support					0.355	7.503	−0.309	−7.077
Self-efficacy							−0.390	−8.897
R^2^	0.306		0.284		0.383		0.544	
△R^2^	0.302		0.280		0.354		0.539	
F	168.053		150.905		110.285		151.274	

The Bootstrap method with 5,000 resamples was used to examine the mediating effects of family support and self-efficacy on the relationship between physical activity and social anxiety (Table 7). The results showed that total effect of physical activity on social anxiety was significant (β_total = −0.547; 95% CI [−0.631, −0.464]). By contrast, in the full mediation model including family support and self-efficacy, the direct effect was not significant (β_direct = −0.181; 95% CI [−0.266, 0.097]), indicating that the association operates primarily through indirect pathways (bias-corrected bootstrap, 5,000 resamples). The direct effect accounted for 33.14% of the total effect.

**Table 7:** Mediating effect of family support and self-efficacy.

Effect	Path	Effect value	Standard error	LLCL	ULCL	Effect ratio
Total effect		−0.547	0.424	−0.638	−0.464	100%
Direct effect	Direct path	−0.181	0.043	−0.266	−0.097	33.09%
Total indirect effect		−0.366	0.037	−0.441	−0.296	66.91%
Indirect effect	Path 1	−0.165	0.026	−0.219	−0.117	30.16%
	Path 2	−0.128	0.024	−0.178	−0.086	23.40%
	Path 3	−0.073	0.014	−0.102	−0.048	13.35%

**Figure 1:**
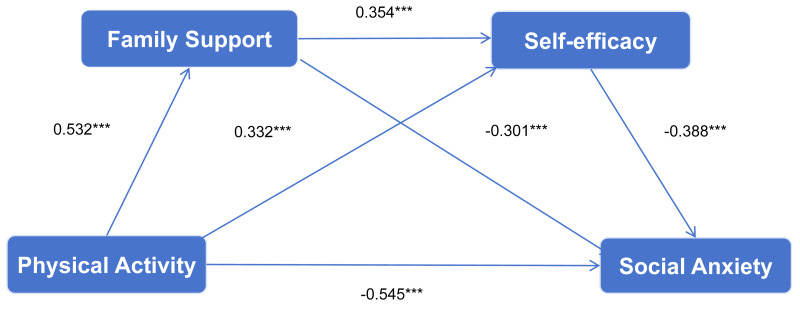
Chain mediating effect model of family support and self-efficacy.

The mediation model indicated that physical activity significantly predicted social anxiety, with family support and self-efficacy serving as significant mediators through three indirect pathways. The total indirect effect was −0.366 (95% bootstrap confidence interval: LLCL = −0.441, ULCL = −0.296), accounting for 66.86% of the total effect. Specifically, the first indirect pathway (Path 1: physical activity → family support → social anxiety) had an effect size of −0.165, accounting for 30.16% of the total effect. The second pathway (Path 2: physical activity → self-efficacy → social anxiety) exhibited an effect size of −0.128, accounting for 23.40% of the total effect. Finally, the third pathway (Path 3: physical activity → family support → self-efficacy → social anxiety), representing the chain mediation effect, yielded an effect size of −0.073, accounting for 13.35% of the total effect. Notably, the chain pathway (Physical activity → Family support → Self-efficacy → Social anxiety) accounted for 13.35% of the total effect, which is smaller than the single-mediator pathways *via* family support (30.16%) and *via* self-efficacy (23.40%), respectively. Thus, hypothesis H4 was supported. The chain mediation model based on these findings is illustrated in Fig. 1.

## Discussion

### Significant negative effect of physical activity on social anxiety among college students

The results of this study indicate a significant negative correlation between physical activity and social anxiety, suggesting that higher levels of physical activity predict lower social anxiety among college students. This pattern is consistent with prior evidence in student populations (McDowell et al., 2019) and with recent quantitative syntheses that specify both magnitude and parameters of effect in university samples. Meta-analytic and network meta-analytic work shows that structured exercise is associated with meaningful improvements in mental health among college students, while large longitudinal cohorts report lower incident anxiety among more physically active individuals, supporting a plausible protective association (Chen et al., 2025; Li et al., 2025). At the same time, critical appraisals emphasize heterogeneity and methodological limitations in anxiety-specific trials, which underscores the value of explanatory, theory-driven models such as the mediator framework tested here.Within educational settings, participation in physical education and sport exposes students to new motor skills and knowledge that are incorporated into their personal repertoire, fostering adaptability. The interpersonal demands of skill acquisition and competition require effective communication with teachers and peers, thereby strengthening interpersonal competence, empathy, and cooperation. Challenges encountered in training and competition can build resilience, improve stress tolerance, and enhance emotion regulation. Moreover, sport contexts emphasize respect, responsibility, and adherence to social norms; these values may generalize beyond sport to support holistic development (Holt et al., 2017). Taken together, these mechanisms provide a credible behavioral and psychosocial basis for the observed association and align with our focus on explanatory pathways evaluated in this study.

### The mediating effect of family support between physical activity and social anxiety among college students

The findings indicate that family support partially mediates the relationship between physical activity and social anxiety among college students, accounting for a relative mediation effect of 30.16%. This suggests that physical activity not only directly reduces social anxiety but also indirectly alleviates it by enhancing family support, thereby confirming Hypothesis 2 of this study. These results align with the psychological mechanism underlying physical activity in social interactions, proposing that participation in physical activity is positively associated with positive interpersonal interactions and contributes to the development of positive emotional experiences. Specifically, during physical activities, individuals engage in conceptual exchanges, interpersonal communications, and emotional interactions, which strengthen their social networks and enhance their ability to obtain various forms of support. Such interactions have beneficial effects on improving individuals’ relational competencies, emotional regulation, and adaptive capacities in stressful situations (Vankim & Nelson, 2013). Consequently, when college students actively participate in physical activities, they have increased opportunities to develop social interaction skills, positively influencing their ability to handle social challenges and effectively reducing social anxiety. For instance, in physical education classes, students frequently interact with peers and instructors, progressively developing effective communication methods and interpersonal skills. This practice enables them to present themselves confidently and comfortably in future social settings.

### The mediating effect of self-efficacy between physical activity and social anxiety among college students

Previous studies have shown that physical activity positively predicts self-efficacy in college students. Consistent with existing research, this study identified self-efficacy as a significant mediator between physical activity and social anxiety. High self-efficacy not only helps individuals maintain psychological health during stress and frustration but also enhances determination, perseverance, and self-control, thus promoting personal growth and overall development. College students who engage in higher levels of physical activity typically possess greater self-efficacy. Consequently, these students display stronger resilience, improved emotional control, persistence, and a more optimistic outlook when facing challenges, thereby better managing emotions and handling interpersonal relationships during social interactions (Bandura, 1997).

### Chain mediating effects of family support and self-efficacy between physical activity and social anxiety among college students

Physical activity was associated with lower social anxiety primarily through indirect effects. After accounting for family support and self-efficacy, the direct path from physical activity to social anxiety was small and not statistically significant based on bootstrap confidence intervals, indicating that the association operates mainly through indirect mechanisms. Among the indirect pathways, the route *via* family support accounted for 30.16% of the total effect and the route *via* self-efficacy accounted for 23.40%, whereas the sequential chain path physical activity → family support → self-efficacy → social anxiety accounted for 13.35%. Thus, in this dataset the single mediator routes were larger in magnitude, while the chain pathway adds mechanistic coherence by illustrating how family context may cultivate efficacy beliefs that, in turn, attenuate social anxiety. These patterns are consistent with recent syntheses and student samples, which identify self-efficacy as a recurrent mediator linking physical activity to mental-health improvements and emphasize the salience of social support and related resilience resources for anxiety reduction (Vicary et al., 2025; White et al., 2024; Yu et al., 2024). From a practical perspective, interventions that directly strengthen family support or self-efficacy are likely to yield larger indirect benefits, with the sequential process offering complementary mechanistic insight.

### Limitations and future directions

This study has several limitations. First, the cross-sectional design precludes causal inference and temporal ordering; mediation paths should be interpreted as associations. Second, the sampling frame (university students in Sichuan, China) constrains generalizability to non-student populations and to regions beyond Sichuan/China. Third, socioeconomic status (*e.g.*, parental education, household income) and family structure (*e.g.*, single- *vs* two-parent households) were not collected and may confound relations involving family support and self-efficacy. Fourth, reliance on single-source self-report may inflate common-method variance. Additionally, although gender, education, and age were controlled in all models, formal multi-group measurement-invariance testing was not conducted; comparability of measurement properties and structural paths across key subgroups therefore remains unverified.

Future work should establish time precedence and mechanisms *via* multi-wave cohorts and randomized trials. A 3–4-wave cohort over 6–12 months with identical measures would enable cross-lagged and longitudinal mediation tests; adding accelerometry and brief ecological momentary assessments could reduce common-method bias. In parallel, a semester-length randomized controlled trial comparing structured moderate-to-vigorous physical activity with an active control (*e.g.*, health education or stretching) should assess outcomes at baseline, mid-intervention, post-intervention, and follow-up, using pre-registered hypotheses, stratification by baseline anxiety and gender, intention-to-treat analyses, and formal tests of measurement invariance and subgroup homogeneity. Replication across multiple provinces and diverse institution types (public/private; comprehensive/specialty; urban/rural) is also recommended.

## Conclusions

Among university students in Sichuan, China, higher physical activity, stronger family support, and greater self-efficacy were each associated with lower social anxiety. Family support and self-efficacy significantly mediated the physical activity–social anxiety relationship. These findings support integrated campus strategies that expand physical-activity opportunities and strengthen family support and self-efficacy; longitudinal and experimental studies are needed to establish causal pathways.

##  Supplemental Information

10.7717/peerj.20511/supp-1 Supplemental Information 1 Raw data

10.7717/peerj.20511/supp-2 Supplemental Information 2 STROBE Checklist

10.7717/peerj.20511/supp-3 Supplemental Information 3 Questionnaire (English)

10.7717/peerj.20511/supp-4 Supplemental Information 4 Questionnaire (Chinese)

## Additional Information and Declarations

### Data Availability

The following information was supplied regarding data availability: The raw data is available in the Supplemental Files. The de-identified dataset and analysis materials supporting the findings of this study are available at Zenodo: zheng, . zhanfei . (2025). Physical Activity–Social Anxiety Study in College Students: Open Dataset and Code [Data set]. Zenodo. https://doi.org/10.5281/zenodo.17096171.
