# Supplementary material for: The impact of physical activity on social anxiety among college students: an analysis of the chain mediation effect of family support and self-efficacy
Source: PeerJ. 2026 Jan 5;14:e20511. doi: 10.7717/peerj.20511 (PMC12782036; doi:10.7717/peerj.20511)
Supplement: Supplemental Information 1 [file peerj-14-20511-s001.zip › raw data/Codebook.docx]

Variable: SEX

Values:

1 = Male

2 = Female

Variable: AGE

Values:

1 = 18-20

2 = 21-23

3 = 24-26

4 = above 26

Variable: DEGREE

Values:

1 = Undergraduate

2 = Master
