# Supplementary material for: The impact of physical activity on social anxiety among college students: an analysis of the chain mediation effect of family support and self-efficacy
Source: PeerJ. 2026 Jan 5;14:e20511. doi: 10.7717/peerj.20511 (PMC12782036; doi:10.7717/peerj.20511)
Supplement: Supplemental Information 2 [file peerj-14-20511-s002.pdf]

STROBE Statement—checklist of items that should be included in reports of observational studies

	Item No.	Recommendation	Page No.	Relevant text from manuscript
**Title and abstract**	1	(*a*) Indicate the study's design with a commonly used term in the title or the abstract	Page 1	The study design is explicitly indicated in the abstract with the phrase "a cross-sectional survey", making it clear that this is a cross-sectional study.
		(*b*) Provide in the abstract an informative and balanced summary of what was done and what was found	Page 1	The abstract provides a concise and balanced overview: it states the objective (to examine the mediating roles of family support and self-efficacy between physical activity and social anxiety), describes the method (cross-sectional survey of 399 college students using SEM and bootstrap), summarizes the main results (negative associations, significant mediating effects), and presents the conclusion (physical activity alleviates social anxiety through these mediators). This aligns with STROBE's requirement for an informative and balanced abstract.
**Introduction**				
Background/rationale	2	Explain the scientific background and rationale for the investigation being reported	Pages 2–3	The Introduction explains that

				social anxiety is prevalent among college students and negatively impacts their well-being. It further reviews existing literature indicating that physical activity can alleviate anxiety and enhance self-efficacy and family support. The rationale is to examine how these mediators function in the relationship between physical activity and social anxiety.
Objectives	3	State specific objectives, including any prespecified hypotheses	Page 3	The Introduction clearly states the study’s objective: to examine whether physical activity reduces social anxiety through the mediating roles of family support and self-efficacy. Four hypotheses are presented: H1 – direct negative effect of physical activity on social anxiety; H2 & H3 – mediation by family support and self-efficacy; H4 – chain mediation effect.
**Methods**				
Study design	4	Present key elements of study design early in the paper	Page 3–4	The Methods section introduces the study as a cross-sectional survey using stratified random sampling among university students in Sichuan Province.

				Data were collected via standardized questionnaires and analyzed using SPSS and AMOS. This description appears at the beginning of the Methods section.
Setting	5	Describe the setting, locations, and relevant dates, including periods of recruitment, exposure, follow-up, and data collection	Page 3–4	The study was conducted among undergraduate and graduate students in multiple universities in Sichuan Province, China. Data were collected via anonymous online questionnaires distributed across strata. Although the exact dates are not specified, the setting and recruitment method are clearly described.
Participants	6	(*a*) *Cohort study*—Give the eligibility criteria, and the sources and methods of selection of participants. Describe methods of follow-up<br>*Case-control study*—Give the eligibility criteria, and the sources and methods of case ascertainment and control selection. Give the rationale for the choice of cases and controls<br>*Cross-sectional study*—Give the eligibility criteria, and the sources and methods of selection of participants	Page 3–4	The study used stratified random sampling to recruit undergraduate and graduate students from various universities in Sichuan Province. Inclusion criteria included current student status; participants completed an online anonymous questionnaire. Of the 399 collected, 391 valid responses were retained after excluding patterned or incomplete responses.
		(*b*) *Cohort study*—For matched studies, give matching criteria and number of exposed and		

		unexposed<br>*Case-control study*—For matched studies, give matching criteria and the number of controls per case		
Variables	7	Clearly define all outcomes, exposures, predictors, potential confounders, and effect modifiers. Give diagnostic criteria, if applicable	Pages 4–5	The study clearly defines:<br>－ Outcome variable: Social anxiety, measured using the IAS scale.<br>－ Exposure: Physical activity, measured via the PARS-3 scale.<br>－ Mediators: Family support (PSS-Fa) and self-efficacy (GSES).<br>－ Covariates: Gender, age, and education level (controlled for in regression models).<br>Each variable's conceptual role and measurement are explicitly described.
Data sources/ measurement	8*	For each variable of interest, give sources of data and details of methods of assessment (measurement). Describe comparability of assessment methods if there is more than one group	Pages 4–5	All variables were measured using validated Chinese versions of established self-report scales:<br>－ Physical activity: PARS-3<br>－ Social anxiety: IAS (Peng et al. revision)<br>－ Family support: PSS-Fa (adapted for Chinese students)<br>－ Self-efficacy: General Self-Efficacy Scale (GSES)<br>Each scale's item format, scoring rules, reliability (Cronbach's α), and validation

				references are detailed. All data were collected using the same online platform across participants, ensuring measurement comparability.
Bias	9	Describe any efforts to address potential sources of bias	Page 5	The study addresses common method bias (CMB) by conducting Harman's single-factor test, a widely used technique in self-report survey research. Results showed that the first factor explained only 31.9% of variance—below the 40% threshold—indicating no serious bias from common method variance.
Study size	10	Explain how the study size was arrived at	Page 3–4	The study reports that 399 questionnaires were distributed, and after screening for patterned responses, incomplete data, and short answering times, 391 valid questionnaires were retained (effective response rate: 97.99%). No a priori sample size calculation was mentioned, but the final sample was deemed sufficient for the planned structural equation modeling and bootstrapping analysis.

Continued on next page

Quantitative variables	11	Explain how quantitative variables were handled in the analyses. If applicable, describe which groupings were chosen and why	Pages 4–6	Quantitative variables were treated as continuous in most analyses. Descriptive statistics (mean, SD), Pearson correlations, and structural equation modeling (SEM) were used.<br>For the physical activity variable, the PARS-3 score was categorized into three levels (⩽19 = low, 20 – 42 = medium, ⩾43 = high) based on validated cut-off points, as described in the instrument section. These categories were used for descriptive purposes.
Statistical methods	12	(*a*) Describe all statistical methods, including those used to control for confounding	Pages 5–6	The study used SPSS 26.0 and AMOS 26.0 for data analysis. Techniques included descriptive statistics, Pearson correlation analysis, structural equation modeling (SEM), and bootstrapping (5,000 iterations). Gender, age, and education were entered as covariates in mediation models to control for confounding.
		(*b*) Describe any methods used to examine subgroups and interactions	Page 6	No subgroup or interaction analyses were conducted. This is stated in the paper.
		(*c*) Explain how missing data were addressed	Page 3	The study reports that incomplete or patterned questionnaires were excluded during data cleaning. Final analysis included only

				complete responses (N = 391).
		(*d*) *Cohort study*—If applicable, explain how loss to follow-up was addressed<br>*Case-control study*—If applicable, explain how matching of cases and controls was addressed<br>*Cross-sectional study*—If applicable, describe analytical methods taking account of sampling strategy	Page 3-4	Although stratified random sampling was used for participant recruitment, no weighting or sampling adjustment was applied in the analysis.
		(*e*) Describe any sensitivity analyses	Not applicable	No sensitivity analyses were conducted or reported.
**Results**				
Participants	13*	(a) Report numbers of individuals at each stage of study—eg numbers potentially eligible, examined for eligibility, confirmed eligible, included in the study, completing follow-up, and analysed	Page 3	The study reports that 399 questionnaires were distributed. After screening, 8 invalid responses were excluded, leaving 391 valid participants for analysis.
		(b) Give reasons for non-participation at each stage	Page 3	Reasons for exclusion included patterned answering, incomplete information, and excessively short completion times.
		(c) Consider use of a flow diagram	Not applicable	No flow diagram was provided in the manuscript.
Descriptive data	14*	(a) Give characteristics of study participants (eg demographic, clinical, social) and information on exposures and potential confounders	Pages 3–4, Table 1	Table 1 presents demographic characteristics (gender, education level, age group) of participants. Text sections describe exposure (physical activity) and potential confounders (e.g., age, gender, education) used in regression models.
		(b) Indicate number of participants with missing data for each variable of interest	Page 3	The manuscript does not report missing data for key variables, and only valid questionnaires were

				analyzed. Therefore, it can be inferred that no missing data were included in the final analysis.
		(c) *Cohort study*—Summarise follow-up time (eg, average and total amount)		
Outcome data	15*	*Cohort study*—Report numbers of outcome events or summary measures over time		
		Case-control study—Report numbers in each exposure category, or summary measures of exposure		
		Cross-sectional study—Report numbers of outcome events or summary measures	Pages 5–7; Tables 2, 4, 5, 6	The study reports summary statistics for the outcome variable (social anxiety), including mean, standard deviation, and its correlation with physical activity, family support, and self-efficacy. Outcome values are presented in both the text and Table 2 & Table 4. Additionally, regression coefficients and SEM path coefficients are reported to quantify the outcome relationships.
Main results	16	(*a*) Give unadjusted estimates and, if applicable, confounder-adjusted estimates and their precision (eg, 95% confidence interval). Make clear which confounders were adjusted for and why they were included	Pages 6–8; Table 5, Table 6	The results report standardized regression coefficients (β) for all paths, with p-values and bootstrapped 95% confidence intervals. Models controlled for age, gender, and education level, which were included as covariates due to their known influence on psychological variables.
		(*b*) Report category boundaries when continuous variables were categorized	Page 4	The PARS-3 physical activity score was categorized into three levels: ≤19 = low, 20 - 42 = moderate, ≥ 43 = high activity. These category

		boundaries were clearly stated in the methods.
	(*c*) If relevant, consider translating estimates of relative risk into absolute risk for a meaningful time period	Not applicable

Continued on next page

Other analyses	17	Report other analyses done—eg analyses of subgroups and interactions, and sensitivity analyses	Page 6	The manuscript states that no subgroup analyses or interaction effects were tested. Similarly, no sensitivity analyses were conducted. The focus was on the hypothesized chain mediation model, tested via SEM with bootstrapping.
**Discussion**				
Key results	18	Summarise key results with reference to study objectives	Page 8	The Discussion section summarizes that physical activity is significantly and negatively associated with social anxiety, both directly and indirectly via family support and self-efficacy. All four hypotheses (H1–H4) are supported by the findings, confirming the proposed chain mediation model. The summary links clearly back to the study's original objectives.
Limitations	19	Discuss limitations of the study, taking into account sources of potential bias or imprecision. Discuss both direction and magnitude of any potential bias	Page 9	The Limitations section discusses several key issues:<br>The cross-sectional design limits causal inference;<br>The sample is geographically limited to Sichuan Province, reducing generalizability;<br>Other potential mediators (e.g., personality traits, peer support) were not included;<br>External factors (e.g., academic stress, life experiences) were not

				controlled.<br>The possible direction and impact of these limitations on the findings are acknowledged.
Interpretation	20	Give a cautious overall interpretation of results considering objectives, limitations, multiplicity of analyses, results from similar studies, and other relevant evidence	Pages 8–10	The Discussion and Conclusion sections provide a balanced interpretation:<br>－ The results support the hypotheses and are consistent with prior literature on the psychological benefits of physical activity.<br>－ The authors acknowledge that the cross-sectional nature precludes causal claims.<br>－ They note limitations (e.g., regional sample, lack of other mediators) and recommend future longitudinal and experimental research.<br>－ Implications for campus mental health promotion are briefly discussed.
Generalisability	21	Discuss the generalisability (external validity) of the study results	Pages 9	The study acknowledges that its findings are based on a sample of college students solely from Sichuan Province, which may limit external validity.<br>The authors recommend caution in generalizing the results to other regions, and suggest future research with diverse and broader populations to enhance

				generalisability.
**Other information**				
Funding	22	Give the source of funding and the role of the funders for the present study and, if applicable, for the original study on which the present article is based	Page 10	The Acknowledgements section explicitly states that the study received no external funding. No sponsors were involved in the design, data collection, analysis, or publication. This indicates full authorial independence.

*Give information separately for cases and controls in case-control studies and, if applicable, for exposed and unexposed groups in cohort and cross-sectional studies.

Note: An Explanation and Elaboration article discusses each checklist item and gives methodological background and published examples of transparent reporting. The STROBE checklist is best used in conjunction with this article (freely available on the Web sites of PLoS Medicine at http://www.plosmedicine.org/, Annals of Internal Medicine at http://www.annals.org/, and Epidemiology at http://www.epidem.com/). Information on the STROBE Initiative is available at www.strobe-statement.org.
