# Supplementary material for: The impact of physical activity on social anxiety among college students: an analysis of the chain mediation effect of family support and self-efficacy
Source: PeerJ. 2026 Jan 5;14:e20511. doi: 10.7717/peerj.20511 (PMC12782036; doi:10.7717/peerj.20511)
Supplement: Supplemental Information 3 [file peerj-14-20511-s003.pdf]

Impact of Sports Participation on Social Anxiety in College Students

Student Participant Information

Thank you for taking the time to participate in this study. This survey aims to investigate the impact of sports participation on social anxiety among college students. All responses will remain anonymous and are used solely for statistical analysis. There are no right or wrong answers—please respond honestly based on your personal experiences. We appreciate your contribution!

Gender [Single-choice] *

☐ Male
☐ Female

Age [Single-choice] *

☐ 17 years old or younger
☐ 18–20
☐ 21–23
☐ 24–26
☐ 27–29
☐ 30–32
☐ 33 years old or older

Current Educational Status [Single-choice] *

☐ Undergraduate
☐ Master’s
☐ Doctoral

Section 1: Sports Participation Scale

(Adapted from the Physical Activity Rating Scale-3 (PARS-3) by Liang Deqing et al.)

This scale assesses sports participation across three dimensions: intensity, duration, and frequency.

What is the intensity of your physical activity? [Single-choice] *

☐ A. Light exercise (e.g., walking, radio calisthenics, table tennis)
☐ B. Low-intensity non-strenuous exercise (e.g., recreational volleyball, slow jogging, tai chi)
☐ C. Moderate-intensity prolonged exercise (e.g., cycling, running, badminton)
☐ D. High-intensity short-term exercise (e.g., basketball, soccer, swimming sprints)
☐ E. High-intensity prolonged exercise (e.g., marathon training, aerobics, competitive swimming)

How long do you engage in the above activity each session? [Single-choice] *

☐ A. ≤10 minutes
☐ B. 11–20 minutes
☐ C. 21–30 minutes
☐ D. 31–60 minutes
☐ E. >60 minutes

How often do you engage in the above activity per month? [Single-choice] *

☐ A. ≤1 time/month

☐ B. 2–3 times/month

☐ C. 1–2 times/week

☐ D. Daily

☐ E. 3–5 times/week

Which sports do you prefer? [Multi-choice] *

☐ A. Walking/running

☐ B. Traveling/hiking

☐ C. Dance (e.g., ballroom, hip-hop)

☐ D. Ball sports (e.g., basketball, soccer)

☐ E. Skipping rope

☐ F. Martial arts/qigong

☐ G. Gymnastics/strength training

☐ H. Swimming

☐ I. Other (please specify): __________

Section 2: Social Anxiety Scale

(Adapted from the Interaction Anxiousness Scale (IAS) by Leary; Chinese version revised by Peng Chunzi et al.)

Rate how strongly each statement applies to you using the following scale:

1 = Not at all characteristic of me

2 = Slightly characteristic

3 = Moderately characteristic

4 = Very characteristic

5 = Extremely characteristic

I often feel nervous even at informal gatherings.

I usually feel uncomfortable around people I don’t know.

I feel relaxed talking to an opposite-sex person. (R)

I get nervous when talking to teachers or supervisors.

Parties often make me feel anxious and uneasy.

Compared to others, I am less shy in social situations. (R)

I often feel nervous talking to unfamiliar same-sex people.

I get nervous during job interviews.

I wish I were more confident in social settings.

I rarely feel anxious in social situations. (R)

I am a shy person.

I often feel nervous talking to an attractive opposite-sex person.

I feel nervous making phone calls to people I don’t know well.

I get nervous talking to authority figures.

I can usually relax even in groups very different from me. (R)

(Note: Items marked with (R) are reverse-scored.)

Section 3: Family Support Scale

(Adapted from the Family Support Scale (PSS-Fa) by Procidano & Heller)
Answer "Yes" or "No" based on your experience:

My family unconditionally supports me when I need help.
I receive good advice from my family members.
My family relationships are less close than those with others.
After sharing pain with a close family member, they share my distress.
My family listens to my thoughts willingly.
I share many joys with my family.
I rely on my family for emotional support.
I can talk to a family member about feeling down without being ridiculed.
My family and I openly discuss shared concerns.
My family responds sensitively to my needs.
My family helps me solve problems.
I have good relationships with many family members.
Talking to my family makes me uncomfortable.
I feel uneasy if others become too close to my family.
I wish my family were different from how it is now.

Section 4: General Self-Efficacy Scale (GSES)

(Chinese version adapted by Wang Caikang et al., 2001)
Rate your agreement with each statement:
1 = Strongly disagree
2 = Disagree
3 = Uncertain
4 = Agree
5 = Strongly agree

If I try hard enough, I can solve any problem.
I can achieve what I want despite opposition.
Staying committed to goals comes easily to me.
I can handle unexpected challenges confidently.
My abilities enable me to manage unforeseen situations.
With effort, I can overcome most difficulties.
I stay calm under pressure because I trust my problem-solving skills.
I can find multiple solutions when facing a problem.
I think of ways to cope when faced with trouble.
I can handle whatever life throws at me.
