# Supplementary material for: The impact of physical activity on social anxiety among college students: an analysis of the chain mediation effect of family support and self-efficacy
Source: PeerJ. 2026 Jan 5;14:e20511. doi: 10.7717/peerj.20511 (PMC12782036; doi:10.7717/peerj.20511)
Supplement: Supplemental Information 4 [file peerj-14-20511-s004.pdf]

运动参与对大学生社交焦虑情况的影响

同学您好，感谢您在百忙之中参与此次调查。本次调查主要为了研究运动参与对大学生社交焦虑情况的影响。本问卷实行匿名制，所有数据只用于统计分析，题目选项无对错之分，请按自己的实际情况填写。占用您的宝贵时间，再次向您表示感谢。

性别 [单选题] *

○男

○女

年龄 [单选题] *

○17 岁及以下

○18-20

○21-23

○24-26

○27-29

○30-32

○33 及以上

现阶段就读情况 [单选题] *

○专科在读

○本科在读

○硕士在读

○博士在读

一、运动参与量表

采用梁德清等人编制的《体育活动等级量表(PARS-3)》。本量表从参加体育锻炼的强度，时间，频率 3 个方面来考虑运动量。

1、您进行体育运动的强度怎样?（ ） [单选题] *

○A.轻微运动（如散步、做广播体操、打门球等）

○B.小强度的不太紧张的运动（如消遣娱乐性的打排球、乒乓球、慢跑、打太极拳等）

○C.中等强度较激烈持久的运动（如骑自行车跑步打乒乓球等）

○D.呼吸急促、出汗很多的大强度的，但不持久的运动（如打羽毛球、篮球、网球、足球等）

○E.呼吸急促，出汗很多的大强度的持久的运动（如赛跑、成套健美操练习、游泳等）

2、您在进行上述强度体育活动时，一次多少分钟?（ ） [单选题] *

○A.10 分钟以下

○B.11 至 20 分钟

○C.21 至 30 分钟

○D.31 至 59 分钟

○E.60 分钟以上

3、您一个月进行几次上述体育活动?（ ） [单选题] *

○A.一个月 1 次以下

○B.每周 3 至 5 次

○C.一个月 2 至 3 次

○D.大约每天 1 次

○E.每周 1 到 2 次

4、您喜欢什么项目的体育运动?（ ）

[多选题] *

□A.散步、跑步

□B.旅游、郊游

□C.体育舞蹈

□D.球类

□E.跳绳

□F.太极拳、养生术

□G.健身器活动

□H.游泳

□I.其他

二、社交焦虑量表

采用 Leary 编制的交往焦虑量表(Interaction Anxiousness Scale, IAS),中文版由彭纯子等修订。请认真阅读下面的每个条目，并决定其陈述对你适用或其真实的程度。根据以下标准在相应的条目下面的空格中进行选择。

1.即使在非正式的聚会上，我也常感到紧张。 [单选题] *

○a.一点也不相符

○b.与我有一点相符

○c.与我中等强度相符

○d.与我非常相符

○e.与我极其相符

2.与一群不认识的人在一起时，我通常感到不自在。 [单选题] *

○a.一点也不相符

○b.与我有一点相符

○c.与我中等强度相符

○d.与我非常相符

○e.与我极其相符

3.在与一位异性交谈时我通常感到轻松。（R） [单选题] *

○a.一点也不相符

○b.与我有一点相符

○c.与我中等强度相符

○d.与我非常相符

○e.与我极其相符

4.在必须同老师或上司谈话时，我感到紧张。 [单选题] *

○a.一点也不相符

○b.与我有一点相符

○c.与我中等强度相符

○d.与我非常相符

○e.与我极其相符

5.聚会常会使我感到焦虑及不自在。 [单选题] *

○a.一点也不相符

○b.与我有一点相符

○c.与我中等强度相符

○d.与我非常相符

○e.与我极其相符

6.与大多数人相比，我在社会交往中可能较少羞怯(R) [单选题] *

○a.一点也不相符

○b.与我有一点相符

○c.与我中等强度相符

○d.与我非常相符

○e.与我极其相符

7.在与我不太熟悉的同性谈话时，我常常感到紧张。 [单选题] *

○a.一点也不相符

○b.与我有一点相符

○c.与我中等强度相符

○d.与我非常相符

○e.与我极其相符

8.在求职面试时我会紧张的。 [单选题] *

○a.一点也不相符

○b.与我有一点相符

○c.与我中等强度相符

○d.与我非常相符

○e.与我极其相符

9 我希望自己在社交场合中信心更足一些。 [单选题] *

○a.一点也不相符

○b.与我有一点相符

○c.与我中等强度相符

○d.与我非常相符

○e.与我极其相符

10.在社交场合中，我很少感到焦虑。(R) [单选题] *

○a.一点也不相符

○b.与我有一点相符

○c.与我中等强度相符

○d.与我非常相符

○e.与我极其相符

11 一般而言，我是一个害羞的人。 [单选题] *

○a.一点也不相符

○b.与我有一点相符

○c.与我中等强度相符

○d.与我非常相符

○e.与我极其相符

12.在与一位迷人的异性交谈时我经常感到紧张。 [单选题] *

○a.一点也不相符

○b.与我有一点相符

○c.与我中等强度相符

○d.与我非常相符

○e.与我极其相符

13.给不太熟的人打电话时我通常觉得紧张。 [单选题] *

○a.一点也不相符

○b.与我有一点相符

○c.与我中等强度相符

○d.与我非常相符

○e.与我极其相符

14.我在与权威人士谈话时感到紧张。 [单选题] *

○a.一点也不相符

○b.与我有一点相符

○c.与我中等强度相符

○d.与我非常相符

○e.与我极其相符

15.即使处于一群和我相当不同的人群之中，通常我仍感到放松。(R) [单选题] *

○a.一点也不相符

○b.与我有一点相符

○c.与我中等强度相符

○d.与我非常相符

○e.与我极其相符

三、家庭支持量表

家庭支持自评量表（Family Support Scale,PSS-Fa）美国 Procidano 和 Heller 根据我国国情对家庭支持自评量表进行了修订。根据实际情况选择是或否。

1. 当我需要的时候，我的家庭会给我义不容辞的支持。 [单选题] *

○是

○否

2. 我能得到家庭成员给我的好主意。 [单选题] *

○是

○否

3. .我与家庭成员之间的亲密关系不如与其他人的关系好。 [单选题] *

○是

○否

4. 我向家中最亲密的成员倾诉痛苦后，他(她)也感到同样痛苦。 [单选题] *

○是

○否

5. 家庭成员乐于倾听我的想法。 [单选题] *

○是

○否

6. 我的许多快乐都与家庭成员共同分享。 [单选题] *

○是

○否

7. 我依赖家庭成员给我情绪(精神)支持。 [单选题] *

○是

○否

8. 如果我感到沮丧，我能向某个家庭成员诉说，且以后不会被嘲笑。 [单选题] *

○是

○否

9. 我与家庭成员之间所考虑的共同的事情能彼此公开交流。 [单选题] *

○是

○否

10. 对我个人的需要，我的家庭成员反应敏感。 [单选题] *

○是

○否

11. 家庭成员乐于帮我解决问题。 [单选题] *

○是

○否

12. 我和许多家庭成员之间关系很好。 [单选题] *

○是

○否

13. 当我向家庭成员倾诉时，使我感到不舒服。 [单选题] *

○是

○否

14. 如果其他人和家庭成员之间的关系过于密切，我感到不舒服。 [单选题] *

○是

○否

15. 我希望我的家庭与现在有所不同。 [单选题] *

○是

○否

四、自我效能理论量表

一般自我效能感量表(GSES)由 Schwarzer 等人编制，中文版由王才康等人（2001）翻译修订。请根据自我情况进行选择。

1.如果我尽力去做的话，我总是能够解决问题的 [单选题] *

○极不符合

○不符合

○不确定

○符合

○极符合

2.即使别人反对我，我仍有办法取得我所要的 [单选题] *

○极不符合

○不符合

○不确定

○符合

○极符合

3.对我来说，坚持理想和达成目标是轻而易举的 [单选题] *

○极不符合

○不符合

○不确定

○符合

○极符合

4.我自信能有效地应付任何突如其来的事情 [单选题] *

○极不符合

○不符合

○不确定

○符合

○极符合

5.以我的才智，我定能应付意料之外的情况 [单选题] *

○极不符合

○不符合

○不确定

○符合

○极符合

6.如果我付出必要的努力，我一定能解决大多数的难题 [单选题] *

○极不符合

○不符合

○不确定

○符合

○极符合

7.我能冷静地面对困难，因为我可信赖自己处理问题的能力 [单选题] *

○极不符合

○不符合

○不确定

○符合

○极符合

8.面对一个难题时，我通常能找到几个解决方法 [单选题] *

○极不符合

○不符合

○不确定

○符合

○极符合

9.有麻烦的时候，我通常能想到一些应付的方法 [单选题] *

○极不符合

○不符合

○不确定

○符合

○极符合

10.无论在我身上发生什么事，我都能应付自如 [单选题] *

○极不符合

○不符合

○不确定

○符合

○极符合
